# Blood-Brain Barrier Alterations Provide Evidence of Subacute Diaschisis in an Ischemic Stroke Rat Model

**DOI:** 10.1371/journal.pone.0063553

**Published:** 2013-05-10

**Authors:** Svitlana Garbuzova-Davis, Maria C. O. Rodrigues, Diana G. Hernandez-Ontiveros, Naoki Tajiri, Aric Frisina-Deyo, Sean M. Boffeli, Jerry V. Abraham, Mibel Pabon, Andrew Wagner, Hiroto Ishikawa, Kazutaka Shinozuka, Edward Haller, Paul R. Sanberg, Yuji Kaneko, Cesario V. Borlongan

**Affiliations:** 1 Center of Excellence for Aging & Brain Repair, University of South Florida, Morsani College of Medicine, Tampa, Florida, United States of America; 2 Department of Neurosurgery and Brain Repair, University of South Florida, Morsani College of Medicine, Tampa, Florida, United States of America; 3 Department of Molecular Pharmacology and Physiology, University of South Florida, Morsani College of Medicine, Tampa, Florida, United States of America; 4 Department of Pathology and Cell Biology, University of South Florida, Morsani College of Medicine, Tampa, Florida, United States of America; 5 Department of Internal Medicine, Ribeirão Preto School of Medicine, University of Sao Paulo, Sao Paulo, Brazil; 6 Department of Psychiatry, University of South Florida, Morsani College of Medicine, Tampa, Florida, United States of America; 7 Department of Integrative Biology, University of South Florida, Tampa, Florida, United States of America; University of Regensburg, Germany

## Abstract

**Background:**

Comprehensive stroke studies reveal diaschisis, a loss of function due to pathological deficits in brain areas remote from initial ischemic lesion. However, blood-brain barrier (BBB) competence in subacute diaschisis is uncertain. The present study investigated subacute diaschisis in a focal ischemic stroke rat model. Specific focuses were BBB integrity and related pathogenic processes in contralateral brain areas.

**Methodology/Principal Findings:**

In ipsilateral hemisphere 7 days after transient middle cerebral artery occlusion (tMCAO), significant BBB alterations characterized by large Evans Blue (EB) parenchymal extravasation, autophagosome accumulation, increased reactive astrocytes and activated microglia, demyelinization, and neuronal damage were detected in the striatum, motor and somatosensory cortices. Vascular damage identified by ultrastuctural and immunohistochemical analyses also occurred in the contralateral hemisphere. In contralateral striatum and motor cortex, major ultrastructural BBB changes included: swollen and vacuolated endothelial cells containing numerous autophagosomes, pericyte degeneration, and perivascular edema. Additionally, prominent EB extravasation, increased endothelial autophagosome formation, rampant astrogliosis, activated microglia, widespread neuronal pyknosis and decreased myelin were observed in contralateral striatum, and motor and somatosensory cortices.

**Conclusions/Significance:**

These results demonstrate focal ischemic stroke-induced pathological disturbances in ipsilateral, as well as in contralateral brain areas, which were shown to be closely associated with BBB breakdown in remote brain microvessels and endothelial autophagosome accumulation. This microvascular damage in subacute phase likely revealed ischemic diaschisis and should be considered in development of treatment strategies for stroke.

## Introduction

Stroke is the fourth leading cause of death in the USA [1], contributing to almost 130,000 fatalities [2] and 5.5 million worldwide yearly [3]. Strokes occur due to interruption of blood flow to the brain and are broadly typed by cause as ischemic or hemorrhagic. Approximately 80% of strokes are ischemic [4]. Due to limited treatment options for stroke and likely ongoing cerebral vascular pathology, more than 18% of patients surviving initial stroke suffer another stroke within five years [5]. About 500 of every 100,000 people live with consequences of stroke [6].

Minutes after ischemic stroke insult, dramatic cerebral pathological changes occur at cellular and molecular levels [7], [8]). Ischemic stroke insults can be focal, global, permanent, or transient, primarily leading to deprivations of oxygen, glucose, and essential nutrients in post-ischemic areas. Within the ischemic core and the penumbra, cascades of pathogenic events evolve over time. The complexity and heterogeneity of mechanisms underlying post-ischemic brain injury make it difficult to develop effective therapeutic approaches for stroke.

One of the important aspects in the pathophysiology of ischemic stroke concerns the role of the blood-brain barrier (BBB). Following ischemic insult, cerebral vascular perturbations lead to BBB damage [9]–[13]. In acute ischemic stroke patients, BBB permeability determined with perfusion-CT was noted in ischemic brain tissues within 12 hours after symptom onset [14]. In a rat middle cerebral artery occlusion (MCAO) model of focal permanent or transient ischemia, BBB disruption was exacerbated after reperfusion and correlated with amount of cerebral blood flow [15]. It has been shown that BBB permeability increased in the ischemic mouse hemisphere one hour after reperfusion [11] or between 3 and 5 hours following MCAO in rats [9], [16]. Interestingly, progressive crossing of a small amino acid tracer through the BBB has been seen up to 6 hours post MCAO [17]. However, widespread BBB openings have been noted shortly after ischemia along with delayed openings of the BBB between 6 and 24 hours after forebrain ischemia in rats [18]. Although this and other [9], [12], [19] studies showed biphasic (“open-close-open”) BBB leakage separated by a refractory period in ischemic-reperfusion injury, subsequent results demonstrated an open BBB persisting for up to 4–5 weeks [20], [21]. This long-lasting BBB opening, which occurred in early acute ischemia, might have extended the ischemic insult or the severity of ischemic tissue injury could have served as a key factor influencing the magnitude of post-ischemic BBB leakage [20]. Since increased BBB permeability is often associated with brain edema and swelling [22], [23], BBB leakage may be a serious and even life threatening clinical complication of cerebral ischemia. On the other hand, spontaneous hemorrhagic transformation in ischemic stroke might be a further consequence of increased BBB permeability (reviewed in [24]).

Despite intensive investigations of BBB integrity and pathogenic processes in ischemic stroke, examinations have mostly been limited to the acute phase and the cerebral hemisphere of initial ischemic insult. Changes in blood flow and metabolism were determined over time in the hemisphere contralateral to unilateral cerebral ischemia, identifying the existence of transhemispheric diaschisis [25]. Since then, comprehensive studies focused on certain brain deficits remote from initial (focal) ischemic lesion have characterized diaschisis phenomena in detail [26]–[29]. Crossed cerebellar, thalamic, and cortical diaschisis [30]–[33] are now well recognized at acute, subacute, and chronic post-stroke stages in correlation with clinical and recovery-related outcomes in patients. Remote alterations of blood flow and/or metabolism in contralateral brain regions were reported up to 14 days after cerebral stroke insult in humans [34], [35]. These long-lasting oxygen and nutritional deprivations might significantly affect brain function. In this context, contralateral excitability changes determined widely in the neocortex at 7 and 28 days after transient MCAO in rats [36], [37] likely contribute to functional deficit. This remote excitability effect, known as transcortical diaschisis [36], has been suggested to result from widespread degeneration of corticostriatal connections.

However, less attention has been paid to BBB competence and related pathogenic processes in remote brain areas at subacute ischemic stage. The necessities of such subacute investigations are at least twofold: first, that functional insufficiency as well as improvement after stroke is strongly dependent on vascular perturbations related to BBB integrity and second, the severity of acute ischemic insult might contribute to remote post-acute effects. Thus, the subacute condition, as the transition period between acute and chronic, might influence the magnitude of cerebral ischemic diaschisis and might represent a therapeutic target for stroke.

The aim of this study was to characterize subacute diaschisis in a rat model of focal cerebral ischemia. A specific focus was analyzing BBB condition and pathogenic processes in the contralateral cerebral hemisphere, remote brain structures not directly affected by ischemia.

## Materials and Methods

### Ethics Statement

All described procedures were approved by the Institutional Animal Care and Use Committee at USF and conducted in compliance with the *Guide for the Care and Use of Laboratory Animals*.

### Animals

All animals used in the study were obtained from The Jackson Laboratory, Bar Harbor, Maine. Twenty eight Sprague Dawley adult male rats weighting 265.2±1.49 g were randomly assigned to one of two groups: MCAO (n = 16) or control (n = 12). All rats were housed in a temperature-controlled room (23°C) and maintained on a 12∶12 h dark: light cycle (lights on at 06∶00 AM). Food and water were available ad libitum.

### Middle Cerebral Artery Occlusion

Stroke surgery was performed via transient middle cerebral artery occlusion (tMCAO) using the intraluminal filament technique as previously detailed [38], [39]. The tip of the filament was customized using a dental cement (GC Corporation, Tokyo, Japan). Body temperature was maintained at 37±0.3°C during the surgical procedures. The midline skin incision was made in the neck with subsequent exploration of the right common carotid artery (CCA), the external carotid artery, and internal carotid artery. A 4-0 monofilament nylon suture (27.0–28.0 mm) was advanced from the CCA bifurcation until it blocked the origin of the middle cerebral artery (MCA). Animals were allowed to recover from anesthesia during MCAO. After 60 minutes of MCAO, animals were re-anesthetized with 1–2% isoflurane in nitrous oxide/oxygen (69%/30%) using a face mask and reperfused by withdrawal of the nylon thread. A midline incision was made in the neck and the right CCA was isolated. The animals were then closed and allowed to recover from anesthesia. We have previously standardized the MCAO model [40]–[43], with stroke animals showing at least 80% reduction in regional cerebral blood flow during the occlusion period as determined by laser Doppler (Perimed). To further ensure similar degree of stroke insults, physiological parameters including PaO2, PaCo2, and plasma pH measurements were monitored, and we found no significant differences in our stroke animals. In addition, animals that did not display a 70% swing bias using the elevated body swing test were excluded [39]. About 90% of animals subjected to this stroke surgery in our hands reached the criteria of reduced cerebral blood flow and biased swing activity.

### Perfusion and Tissue Preparation

Seven days after reperfusion, tMCAO rats and controls were sacrificed under CO_2_ inhalation and perfused transcardially with 0.1 M phosphate buffer (PB, pH 7.2) followed by 4% paraformaldehyde (PFA) in PB solution under pressure control fluid delivery at 85 mm Hg. Rats assayed for Evans Blue extravasation received only PB solution. Evans Blue dye (EB, Aldrich Chemical), 961 Da, was used as a tracer for assessing BBB disruption. tMCAO rats (n = 13) and controls (n = 9) were intravenously injected with 1 ml of 2% EB in saline solution via the jugular vein 30 min prior to perfusion. The surgical procedure was performed in tMCAO and control rats using the same protocol, including exposure to anesthesia, as previously described [44] with only a slight modification (i.e., EB was injected via the jugular vein in the present study). Briefly, after perfusion, rat brains and a small part of the liver were rapidly removed from tMCAO rats (n = 10) and controls (n = 7) for EB extravasation assay as described below. After perfusion of remaining rats receiving an EB injection, their brains were immediately removed, fixed intact in 4% PFA in 0.1 M PB for 24–48 hrs and then cryoprotected in 20% sucrose in 0.1 M PB overnight. Coronal brain tissues were cut at 30 µm in a cryostat, thaw-mounted onto slides, and stored at −20°C for immunohistochemical analysis. Rats assayed for EM analysis (tMCAO, n = 3; control, n = 3) were decapitated and the brains were immediately removed and fixed in 4% PFA in 0.1 M PB for 16–24 hours at 4°C. The next day, brains were cut into 1 mm slices, mapped against a diagram of the whole slice at Bregma level of 0.20–0.48 mm accordingly to [45], and the motor cortex (M1/M2) and striatum (CPu) regions were removed and coded from the slices of both brain hemispheres. Coordinates for ipsi- M1/M2 motor cortex area were about 4 mm ventral and 1.5 mm lateral from the striatum on the coronal section. The same coordinates were applied for removal of striatum and motor cortex tissues from the contralateral hemisphere. Tissues were then fixed overnight in 2.5% glutaraldehyde in 0.1 M PB (Electron Microscopy Sciences, Inc., Hatfield, PA) at 4°C and stored for further EM processing.

### BBB Permeability

The EB extravasation assay was performed as previously described [44]. Briefly, after perfusion, rat brains were divided into right and left hemispheres. Brain and liver tissues were weighed and placed in 50% trichloroacetic acid solution (Sigma). Following homogenization and centrifugation, the supernatant was diluted with ethanol (1∶3) and loaded into a 96 well-plate in triplicates. The extracted dye was measured with a spectrofluorometer (Gemini EM Microplate Spectrofluorometer, Molecular Devices) at excitation of 620 nm and emission of 680 nm. Calculations were based on external standards in the same solvent. The tissue EB content was quantified from a linear standard curve derived from known amounts of the dye and was normalized to tissue weight (µg/g). All measurements were performed by two experimenters blinded to the experiment.

### Electron Microscopy

The BBB structural characteristics were identified in different brain structures using electron microscopy. Since the cortex and striatum are the areas most affected by MCAO [46]–[48], structural integrity of microvessels [49] was analyzed in the motor cortex (M1/M2) and striatum (CPu) of both brain hemispheres. Briefly, tissue samples were post-fixed in 1% osmium tetroxide (Electron Microscopy Sciences, Inc., Hatfield, PA) in 0.1 M PB for 1 hour at room temperature (RT) and then dehydrated in a graded series of acetone dilutions. Tissues were transferred to a 50∶50 mix of acetone and LX112 epoxy resin embedding mix (Ladd Research Industries, Burlington VT) and infiltrated with this mix for 1 hour. The tissues were then transferred to a 100% LX112 embedding mix and infiltrated with fresh changes of the embedding mix. The tissues were further infiltrated overnight in fresh embedding medium at 4°C. On the following day, the tissues were embedded in a fresh change of resin in tissue capsules. The blocks were polymerized at 70°C in an oven overnight. The blocks were trimmed and then sectioned with a diamond knife on an LKB Huxley ultramicrotome. Thick sections cut at 0.35 µm were placed on glass slides and stained with 1% toluidine blue stain. Thin sections were cut at 80–90 nm, placed on copper grids, and stained with uranyl acetate and lead citrate.

### BBB Integrity Analysis

For analysis of BBB ultrastructure, microvessels in the motor cortex and striatum of both ipsilateral and contralateral brain hemispheres were examined by an investigator blinded to the animal groups and photographed with a FEI Morgagni transmission electron microscope (FEI, Inc., Hillsboro, OR), using an Olympus MegaView III digital camera (ResAlta Research Technologies Corp., Golden, CO.) at 60 kV. In addition to the EB extravasation assay described above, vascular EB leakage was analyzed in serial brain and liver tissue sections via immunohistochemistry.

### Immunohistochemistry

Immunohistochemical staining for collagen IV, a component of the basement membrane, was performed to determine the vascular network in the brain. Brain tissues were pre-incubated with 10% normal goat serum (NGS) and 0.3% Triton 100X in phosphate-buffered saline (PBS) for 60 min at RT. Rabbit polyclonal anti-collagen IV antibody (1∶300, Abcam) was applied on tissue slides overnight at 4°C. Next day, the slides were washed in PBS and goat anti-rabbit secondary antibody conjugated to FITC (1∶600, Invitrogen) was applied. Tissue sections were then incubated for 2 hrs at RT. The tissues were rinsed in PBS and then coverslipped with Vectashield containing DAPI (Vector) and examined under an Olympus BX60 epifluorescence microscope for EB leakage in microvessels as indicated by collagen IV staining. In a separate set of brain sections, immunohistochemical staining of astrocytes and microglia was performed. For astrocyte staining, tissues were pre-incubated in blocking solution as described above and then incubated overnight with rabbit polyclonal anti-glial fibrillary acid protein primary antibody (GFAP, 1∶500, Dako) at 4°C. The next day, secondary goat anti-rabbit antibody conjugated to FITC (1∶500, Invitrogen) was applied for 2 hrs. After washing, slides were coverslipped with Vectashield containing DAPI (Vector). For microglial staining, tissues were incubated overnight at 4°C with mouse anti-rat OX-6 antibody (OX-6, 1∶500, BD Pharmingen). After primary antibody incubation, the slides were rinsed in PBS and incubated with biotinylated goat-anti-mouse secondary antibody (1∶200, Vector) for 2 hrs at RT. The tissue was then rinsed in PBS and incubated with avidin-biotin-peroxidase enzyme complex (ABC-Elite kit, Vector), followed by 3,3-diaminobenzidine chromogen (DAB, Pierce). Slides were then dehydrated, coverslipped with mounting medium, and examined under a bright field microscope (Olympus 60X). Immunohistochemical staining for autophagosomes was performed to detect autophagy response. Serial tissue sections of tMCAO and control rat brains were incubated in a blocking solution for 60 min as described above. Rabbit polyclonal anti-Beclin-1 antibody (Beclin-1, 1∶500, Pierce) was applied on the slides overnight at 4°C. The next day, the slides were rinsed in PBS and incubated with secondary goat anti-rabbit antibody conjugated to FITC (1∶500, Molecular Probes) for 2 hrs. After rinsing, slides were coverslipped with Vectashield containing DAPI (Vector) and examined using an Olympus BX60 epifluorescence microscope. Observation of Beclin-1 fluorescent intensity was performed in 5–6 capillaries per examined brain structure in both hemispheres. Diameters of capillaries were measured using Image Pro® Plus (version 4.5.1, Media Cybernetics, Inc., Rockville, MD, USA). The immunohistochemical images of all performed assessments were taken approximately at the same Bregma level analyzed for EM by an investigator blinded to the experiments and animal codes were removed prior to analysis. To avoid bias in the analysis of fluorescence images, specific brain areas were identified in a section using a 10×/0.30 numerical aperture (NA) lens, and then areas of interest were photographed with either a 20×/0.50 NA or 40×/0.75 NA lens, photographing the slide in a random raster pattern. To test for specificity of the immunostaining, the primary antibodies were omitted from control slides. No staining was observed in the control sections.

Additionally, the brain sections were stained with 0.1% Luxol Fast Blue and 0.1% Cresyl Violet technique using a standard protocol for examination of myelin and neuron condition. The Luxol Fast Blue stains myelin sheaths blue, whereas the cresyl violet counterstains the Nissl substance violet.

### Statistical and Semi-quantitative Analysis

For EB extravasation, data are presented as means ± S.E.M. One-way ANOVA with Bonferroni’s Multiple Comparison test (GraphPad Prism, version 5.02, La Jolla, CA, USA) was used. A p<0.05 was considered significant. For semi-quantitative analysis of Beclin-1, GFAP, and OX-6 immunoexpression and Luxol Fast Blue/Cresyl Violet histological staining for myelin and neurons, images were assessed according to a three-point scale: 1 point was assigned to control level (baseline); 2 points for noticeable immunoreactivity or histological staining increase; 3 points for intensive immunoreactivity or histological staining increase; below 1 point was given when less immunoreactivity or histological staining was noted vs. control.

## Results

### Ultrastructure of the Cerebral Microvasculature in Subacute Ischemic Rat Model of tMCAO

The BBB ultrastructure was analyzed in brains of rats sacrificed 7 days after tMCAO using electron microscopy. Structural integrity analysis of microvessels in the striatum and motor cortex of the brain was performed on hemispheres ipsilateral and contralateral to tMCAO damage.

### Striatum

**Figure 1:**
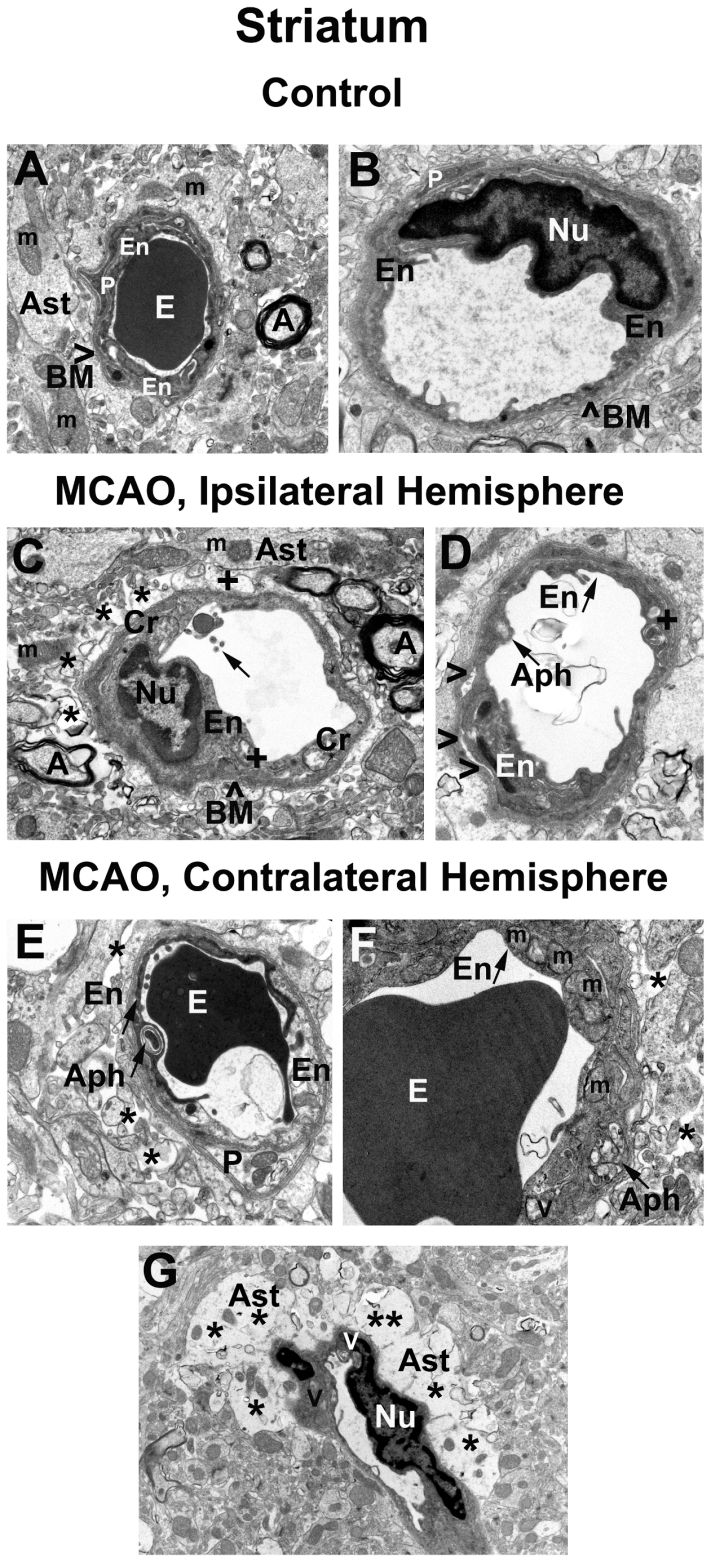
Electron microscope examination of microvasculature in the rat striatum 7 days after MCAO. Representative area of control rat striatum (**A, B**)was characterized by the normal ultrastructural appearance of neurons, capillaries, neuropil, myelinated axons, and surrounding astrocytes. A single layer of endothelial cells (ECs) is surrounded by a single layer of basement membrane (BM), forming an intact BBB. (**C**) In the hemisphere ipsilateral to MCAO insult, ultrastructural abnormalities were observed in striatum capillary endothelia. ECs showed endoplasmic reticulum swelling and formation of numerous large vacuoles in their cytoplasm. Mitochondria in the cytoplasm of most cells showed disruption of cristae. In the lumen of the capillary, fragments of microvilli and mitochondria that have been shed from EC were observed. Near the capillaries were spaces created by degenerating astrocyte cell processes and protein-filled areas. (**D**) A capillary with condensed EC cytoplasm was also observed in ipsilateral striatum. An autophagosome and dilated endoplasmic reticulum and lysosomal membranes were detected in EC. The second EC appeared to be separated from the BM. (**E**) In contralateral striatum, capillaries contain a necrotic EC and a swollen EC. A degenerated pericyte is also apparent. Some areas of edema surround the capillary. A capillary (**F**) with a swollen EC layer containing enlarged mitochondria was determined. Profiles of dilated endoplasmic reticulum and autophagocytic vacuole were observed in EC cytoplasm. Another capillary contained ECs with condensed cytoplasm and vacuoles (**G**). Surrounding the capillary were astrocytes showing severe edema. **En** - endothelial cell, **BM** - basement membrane, **Ast** – astrocyte, **E** – erythrocyte, **m** – mitochondrion, **A** – axon, **V** – vacuole, **P** – pericyte, **Nu** – nucleus, **Aph** – autophagosome, **Cr** – disrupted cristae in the mitochondrion,**+**- swollen endoplasmic reticulum, **arrow** in **C** - broken microvilli, arrowheads in **D** indicate separation of EC from BM. Asterisks in (**C**), (**E**), (**F**), (**G**) indicate extracellular edema. Magnification in (**A**) is 8,900; in (**B**), (**C**), (**D**), (**E**) is 11,000; in (**F**) is 14,000, in (**G**) is 5,600.

The striatum of control rats was characterized by the normal appearance of neurons, capillaries, surrounding astrocytes, and myelinated axons (Figure 1A, B). Capillaries consisted of a single layer of endothelial cells (ECs) surrounded by a layer of basement membrane (BM), sometimes enclosed by additional pericyte cytoplasm and another layer of BM. Organelles in all cells were well preserved and mitochondria showed a normal pattern of cristae.

In the hemisphere ipsilateral to tMCAO, ultrastructural abnormalities were observed in capillary endothelia in striatum (Figure 1C, D). ECs showed endoplasmic reticulum swelling and formation of numerous large vacuoles in their cytoplasm. Mitochondria in the cytoplasm of most cells showed disruption of cristae (Figure 1C). Fragments of microvilli were observed floating free in the capillary lumen, in addition to the obvious disruption of the cristae in the mitochondria of the endothelial cells (Figure 1C). Large autophagosomes were observed in almost all ECs (Figure 1D), with some autophagosomes extending from lumen to basal lamina in attenuated portions of the cells. Near the capillaries were spaces created by degenerating astrocyte cell processes and protein-filled areas formerly occupied by astrocytes. Figure 1D shows a striatum capillary with condensed EC cytoplasm. The EC displayed an autophagosome, while another EC showed profiles of dilated endoplasmic reticulum and lysosomal membranes. The second EC in this capillary appeared to be separating from BM. The thickness of BM was reduced on abluminal sides of damaged ECs (Figure 1D).

Less severe, but still significant, vascular damage was observed in the hemisphere contralateral to tMCAO insult. Capillaries in the striatum (Figure 1E, F, G) had swollen and vacuolated ECs containing numerous autophagosomes. In the contralateral striatum, a capillary contained necrotic EC along with a swollen cell and degenerated pericyte (Figure 1E). Another capillary displayed a swollen EC layer containing enlarged mitochondria and a large autophagosome, which almost ruptured the BM (Figure 1F). Some capillaries contained ECs with condensed cytoplasm and vacuoles (Figure 1G). Surrounding the capillary were astrocytes showing severe edema and a thinning BM.

### Motor Cortex

**Figure 2:**
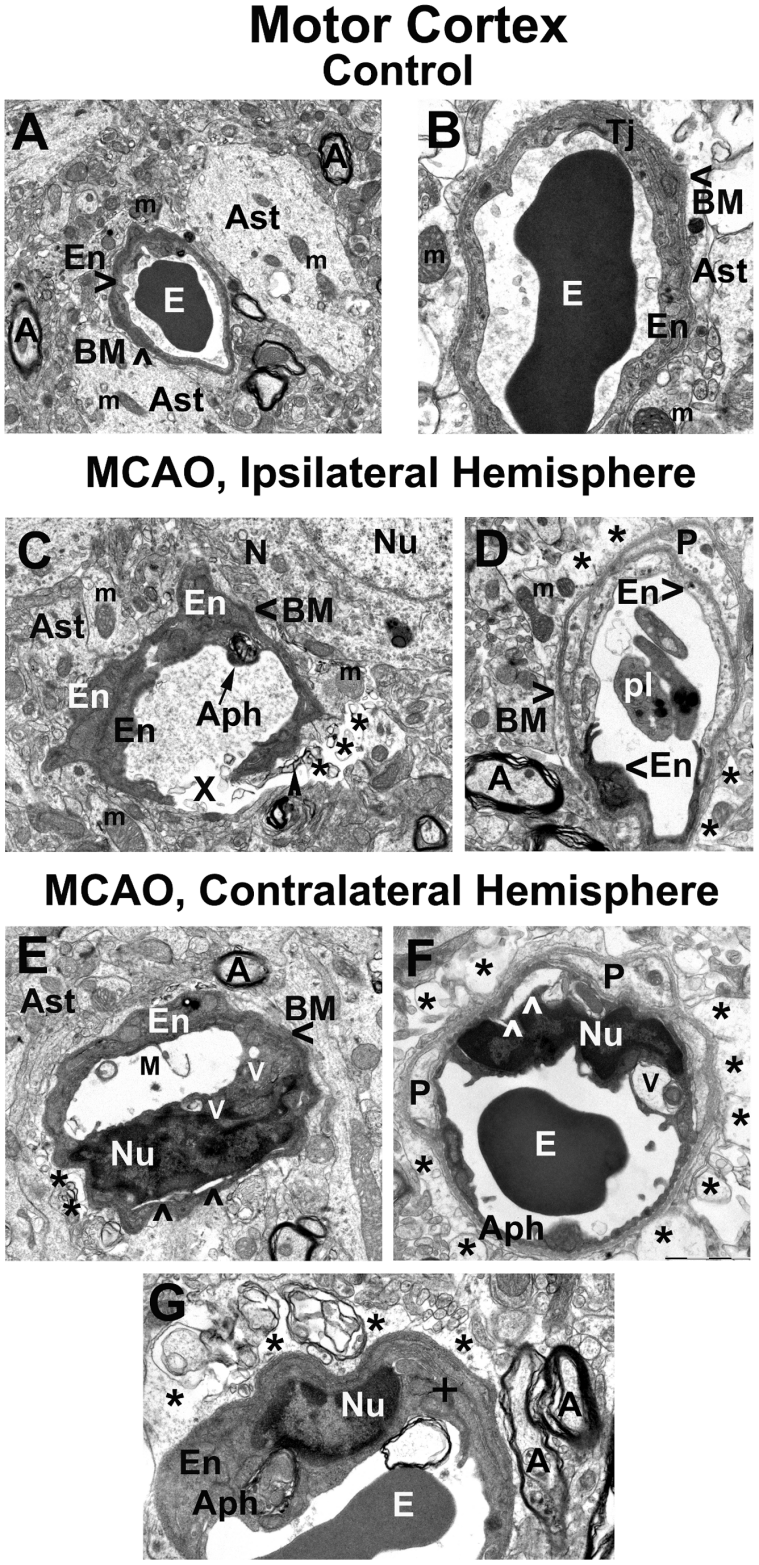
Electron microscope examination of microvasculature in the rat motor cortex 7 days after MCAO. Similar to striatum, capillaries in control motor cortex showed normal ultrastructure consisting of blood vessels with a single endothelium layer, surrounded by BM, astrocyte cell processes, and partially bounded by pericyte cytoplasm (**A, B**). A tight junction was clearly apparent. In the hemisphere ipsilateral to MCAO damage, (**C**) a microaneurysm with a completely ruptured endothelium leading to vascular leakage was detected in the motor cortex. Autophagosome formation in the capillary endothelium may have resulted in capillary rupture. (**D**) Necrotic EC with condensed cytoplasm is adjacent to a healthy EC. Surrounding the capillary are cell processes of a degenerated pericyte. The capillary BM is intact. There is evidence of extracellular edema. Multiple platelets in capillary lumen were detected. (**E**) In motor cortex of the hemisphere contralateral to MCAO insult, the capillary endothelium is swollen and vacuolated. Membrane fragments of endothelial cells were detected in capillary lumen. Dilated endoplasmic reticulum and numerous vacuoles were found in EC cytoplasm. Dilated endoplasmic reticulum in the astrocyte was also determined outside the capillary. Another motor cortex capillary (**F**) displayed one EC lifting off the basement membrane in addition to necrotic EC containing an autophagosome. A large vacuole occupies part of the cell’s cytoplasm. Two degenerated pericytes were seen in the same capillary. Large areas of extracellular edema were observed surrounding the capillary. In a high magnification image (**G**), profiles of dilated endoplasmic reticulum were observed in the EC cytoplasm along with an autophagocytic vacuole, indicating cell stress. Also, a region of extracellular edema surrounded the capillary. **En** - endothelial cell, **BM** - basement membrane, **Ast** – astrocyte, **E** – erythrocyte, **Pl** – platelets, **m** – mitochondrion, **N** - neuron, **A** – axon, **V** – vacuole, **P** – pericyte, **Nu** – nucleus, **Aph** – autophagosome, **X** – ruptured endothelium in a microaneurysm, **M** – endothelial cell membrane fragment in capillary lumen,**+**- swollen endoplasmic reticulum, arrowheads in **F** indicate separation of EC from BM. Asterisks in **(C)**, **(D)**, **(E)**, **(F), (G)** indicate extracellular edema. Magnification in **(A), (E)** is 7,100; in **(C)**, **(D)** is 8,900; in **(F)** is 11,000; in **(B), (G)** is 14,000.

Similar to striatum, capillaries in control motor cortex showed normal ultrastructure consisting of blood vessels with a single endothelium layer, surrounded by BM and partially bounded by pericyte cytoplasm (Figure 2A, B). Astrocyte cell processes were adjacent to the outer capillary surface.

Seven days after tMCAO, in the hemisphere ipsilateral to stroke insult, ultrastructure of capillaries in motor cortex displayed varied aberrations (Figure 2C, D). Swollen ECs, complete pericyte degeneration, and autophagosomes were noted in numerous capillaries. A microaneurysm with a completely ruptured endothelium and BM, leading to vascular leakage, was detected in the motor cortex (Figure 2C). This rupture may have been due to the presence of an autophagosome in the cytoplasm of EC, weakening the endothelial cell and leading to a break of the endothelium and thin BM on the abluminal side. The osmiophilic debris in the area of microvessel damage was likely evidence of a ruptured autophagosome. In numerous motor cortex capillaries, necrotic ECs with condensed cytoplasm were identified adjacent to healthy ECs in the lumen (Figure 2D). Although most parts of the capillary basement membrane in this microvessel were intact, pericyte degeneration was observed. A cluster of platelets was also detected in this capillary lumen. Tissue edema in the extracellular space was revealed at sites of thinner BM (Figure 2D).

In the hemisphere contralateral to tMCAO, capillaries in the motor cortex showed endothelial and pericyte cell damage similar to ultrastructural abnormalities in ipsilateral cortex capillaries (Figure 2E, F, G). In some motor cortex areas, EC membranes appeared ruptured and membrane fragments were detected in capillary lumen (Figure 2E). Autophagic vacuole formation was also observed in the cytoplasm of the endothelial cells. In this microvessel, a long dilated endoplasmic reticulum was seen below the EC nucleus and in an astrocyte cell process. Similarly to the ipsilateral hemisphere, large areas of extracellular edema were observed surrounding the capillary and BM appeared thinner (Figure 2F). Capillary in Figure 2F also displayed EC separating from the BM and a large vacuole occupied part of the cell cytoplasm. Additionally, the inner layer of endothelium consisted of necrotic EC with an autophagosome. Two degenerated pericytes were seen in the same capillary. In a high magnification electron microscopic image (Figure 2G), profiles of dilated endoplasmic reticulum were observed in the EC cytoplasm along with an autophagocytic vacuole, indicating cell stress. Surrounding the capillary was a region of extracellular edema.

Thus, BBB alterations were clearly detected by electron microscopy in the striatum and motor cortex of both ipsi- and contralateral cerebral hemispheres in rats 7 days after tMCAO. Importantly, capillary ultrastructural abnormalities were demonstrated in brain regions distal from the site of primary ischemic injury in subacute phase.

### Microvascular Permeability

**Figure 3:**
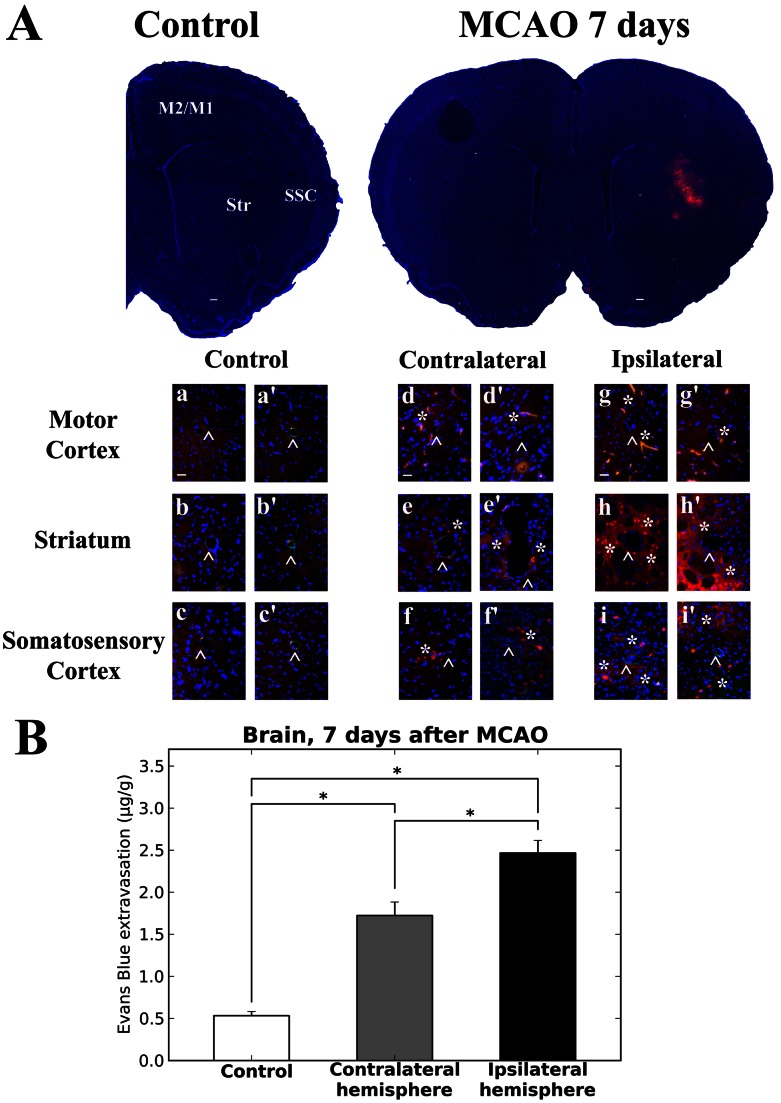
Immunofluorescence and quantitative analysis of Evans Blue extravasation into the rat brain parenchyma 7 days after MCAO. (**A**) In the brains of control rats, EB (red) was clearly detected within capillary lumen of motor cortex (**a, a’**), striatum (**b, b’**), and somatosensory cortex (**c, c’**) using immunofluorescence technique. Collagen IV immunostaining (arrowheads, green) was strongly apparent in basement membrane of vessels. At 7 days after MCAO, significant EB extravasation (asterisks, red) was identified in hemisphere ipsilateral to initial ischemic insult in striatum (**h, h’**), somatosensory (**i, i’**) and motor (**g, g’**) cortices. Extensive EB leakage was also seen in same cerebral structures of contralateral hemisphere (**d–f’**). Diminished or weak immunoexpression for collagen IV (arrowheads, green) was observed in numerous capillaries of both ipsi- and contralateral hemispheres. In full brain images and a–i’, blue color indicates DAPI staining of cell nuclei. Some images contain a purple background due to high density of cell nuclei (blue) and extravasated EB (red). Scale bar in full brain images is 200 µm; in a, d, g is 50 µm (representative for each panel a’–i’). (**B**) Quantitative measurement of tissue EB content showed significantly (p<0.0001) higher extravasated EB levels in ipsi- and contralateral hemispheres vs. control. Significantly (p = 0.0006) elevated EB level was determined in ipsilateral hemisphere compared to contralateral.

Capillary BBB permeability was examined via immunofluorescence technique and quantitative analysis of EB extravasation into the brain parenchyma in tMCAO rats and controls. In the brains of control rats, EB fluorescence was primarily detected as small red dots attached to the capillary lumen of various cerebral structures (Figure 3A, a′, b′, c, c′) due to dye that washed out during perfusion. No EB leakage was observed on the abluminal capillary surface. Collagen IV immunostaining was strongly apparent in all vessels. At 7 days after tMCAO, significant EB extravasation was identified in the hemisphere ipsilateral to initial insult, mostly, in the striatum (CPu), secondary somatosensory and motor cortices (M1/M2) (Figure 3A, g–i′). Extensive EB leakage was also seen in the same cerebral structures of the contralateral hemisphere (Figure 3A, d–f′). Diminished or weak immunoexpression for collagen IV was observed in numerous capillaries of both ipsi- (Figure 3A, g–h′) and contralateral (Figure 3A, d–e′) hemispheres, mostly in striatum and motor cortex. These results correlated with our EM findings showing reduction of BM thickness in some striatum and motor cortex microvessels. Tissue measurements showed significantly higher EB levels in ipsilateral (2.47±0.44 µg/g, p<0.0001) and contralateral (1.72±0.49 µg/g, p<0.0001) hemispheres vs. controls (0.53±0.17 µg/g) (Figure 3B). Significantly (p = 0.0006) elevated EB level was determined in ipsilateral hemisphere compared to contralateral.

Quantification of EB extravasation in the liver, an organ with highly fenestrated capillaries, was performed as control for cerebral EB extravasation. Expected results showed no significant difference in EB extravasation from control (203.91±18.16 µg/g) vs. MCAO (235.95±18.57 µg/g) rat livers.

### Autophagosome Analysis in Cerebral Endothelial Cells

**Figure 4:**
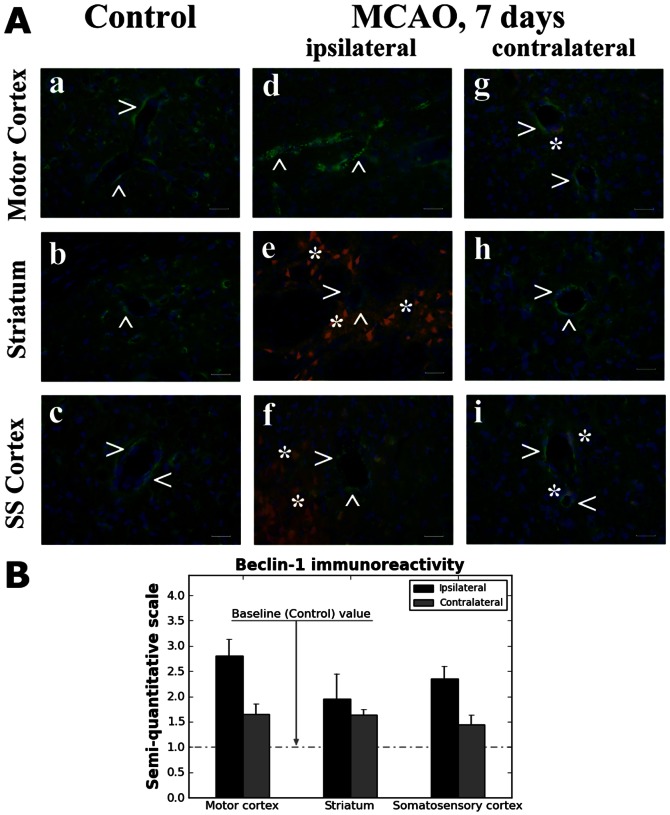
Immunohistochemical analysis of autophagosomes in capillary endothelium in the rat brain 7 days after MCAO. (**A**) Immunofluorescent staining for Beclin-1 (arrowheads, green) showed orderly expression of autophagosomes in capillary endothelium of motor cortex (**a**), striatum (**b**), and somatosensory cortex (**c**) of control rats. Extensive autophagosome accumulation within endothelium in numerous motor cortex, striatum, and somatosensory cortex capillaries of ipsilateral (**d–f**) hemisphere was observed. A slight increase of Beclin-1 fluorescent expression was observed in contralateral capillaries of same brain structures (**g–i**). Scale bar in a-i is 25 µm. (**B**) Semi-quantitative analysis revealed most intensive Beclin-1 immunoexpression in ipsilateral capillaries from all examined brain structures vs. control (baseline). Minor elevation of Beclin-1expression was seen in contralateral capillaries. Note: immunohistochemical analysis of Beclin-1 fluorescent expressions were performed in control, ipsi- and contralateral hemisphere capillaries with the following ranges of diameters: motor cortex −23.1–25.0 µm; striatum –26.0–27.9 µm; somatosensory cortex –22.6–25.8 µm.

Since large autophagosome formations were observed via electron microscopy in numerous ECs followed by rupture and exposure of capillary endothelia in both ipsi- and contralateral hemispheres 7 days after tMCAO, immunohistochemical analysis of Beclin-1 expression was performed in control, ipsi- and contralateral hemisphere capillaries with the following ranges of diameters: motor cortex −23.1–25.02 µm; striatum –26.0–27.9 µm; somatosensory cortex –22.6–25.8 µm. Results showed extensive autophagosome accumulations within ECs in numerous motor cortex (M1/M2), striatum (CPu), and secondary somatosensory cortex capillaries of ipsilateral hemisphere (Figure 4A, d–f) compared to control (Figure 4A, a–c). A slight increase of Beclin-1 fluorescent expression was observed in contralateral capillaries of same brain structures (Figure 4A, g–i) vs. controls. Of note, immunofluorescence expansion of Beclin-1 was noted in both ipsi- and contralateral capillary endothelium of post-stroke animals, likely indicating autophagosome vacuole formation. Semi-quantitative analysis revealed most intensive Beclin-1 immunoexpression in ipsilateral capillaries (Figure 4B).

### Astrocytes and Microglia Analyses

**Figure 5:**
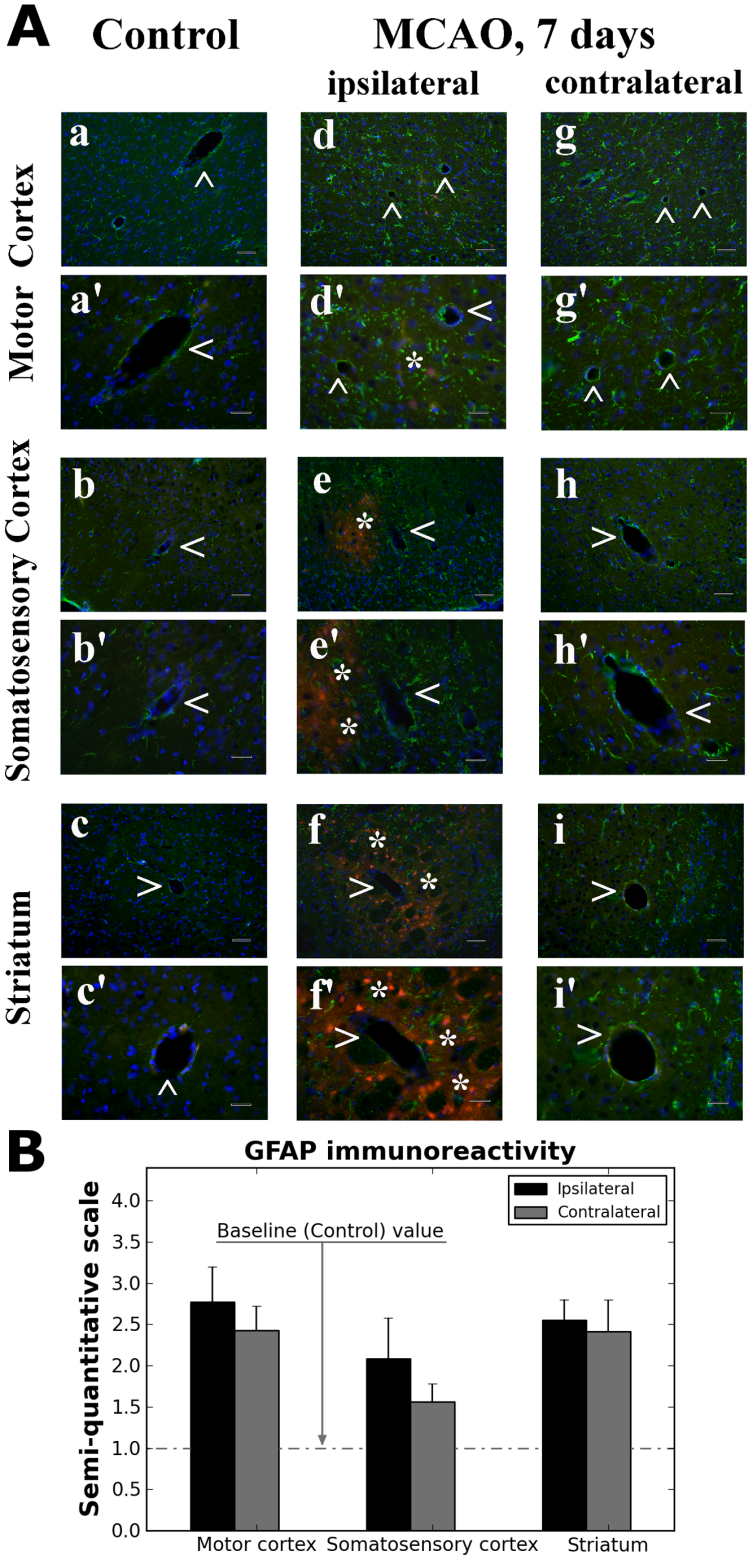
Immunohistochemical analysis of astrocytes. (**A**) Immunohistochemical analysis of astrocytes in the brains of control rats showed normal appearance of cells in the motor cortex (**a, a’**), somatosensory cortex (**b, b’**), and striatum (**c, c’**) parenchyma. GFAP positive cells (green) were distinguished surrounding capillaries (arrowheads) in control brains. In the brains of rats 7 days post-MCAO, astrogliosis was noted in striatum, motor and somatosensory cortices in ipsilateral (**d–f**’) and contralateral (**g–i**′) hemispheres. Alterations of astrocytic end-feet were marked in some capillaries. Asterisks indicate EB leakage (red). Scale bar in a–i is 50 µm, in a’–i’ (higher magnification images of **a–i**) is 25 µm. (**B**) Immunoexpression for GFAP was analyzed semi-quantitatively and higher scores were determined in areas of ipsilateral brain structures vs. control (baseline). Importantly, the same high degree of GFAP expression was noted for ipsi- and contralateral striatum and motor cortex.

Immunohistochemical analysis of astrocytes in the brains of control rats demonstrated normal appearance of cells in the motor cortex (M1/M2), secondary somatosensory cortex, and CPu (striatum) parenchyma (Figure 5A, a–c’). GFAP positive cells were distinguished surrounding capillaries in control brains. In the brains of rats 7 days post tMCAO, astrogliosis, which evidenced EB leakage in ipsilateral (Figure 5A, d–f’) and contralateral (Figure 5A, g–I’) hemispheres, was noted in cerebral structures. Immunoexpression for GFAP was analyzed semi-quantitatively and higher scores were determined in areas of ipsilateral brain structures (Figure 5B). Interestingly, the same high score was noted for ipsi- and contralateral striatum. Alterations of astrocytic end-feet were marked in some capillaries.

**Figure 6:**
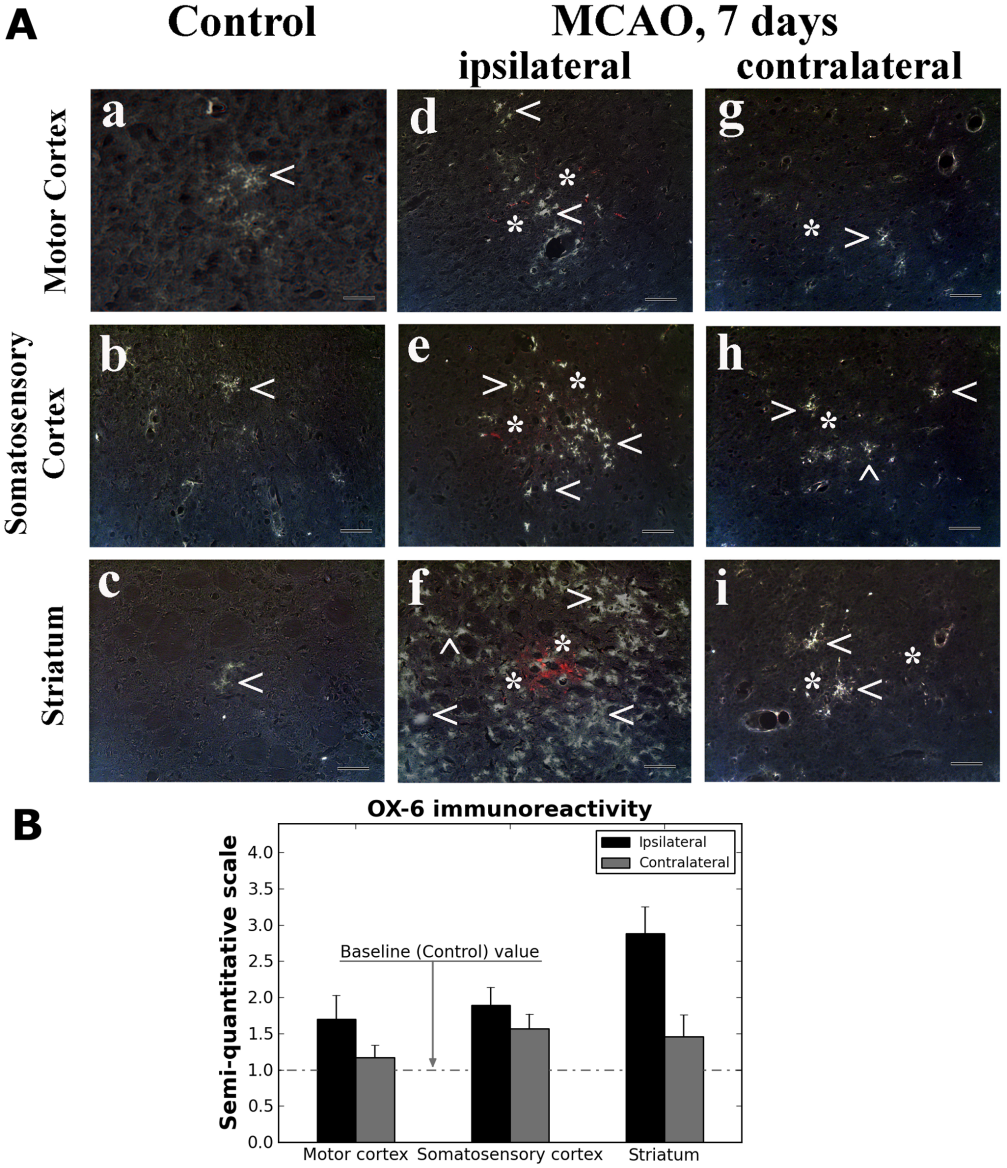
Immunohistochemical analysis of activated microglia. A few OX-6 positive cells (arrowhead) were identified in motor (**a**) and somatosensory (**b**) cortices and striatum (**c**) in control rats. A large number of activated microglial cells were determined in ipsilateral MCAO hemisphere, in brain structures with EB extravasation (**d–f**). Morphologically, these cells were characterized by large cell bodies and short processes. In the contralateral hemisphere, activated microglia were observed, primarily in the striatum (**i**) and somatosensory cortex (**h**). Some OX-6 positive cells were also seen in the contralateral motor cortex (**g**). Arrowheads indicate microglial cells. Asterisks indicate EB leakage (red). Images of OX-6 expression were converted to grayscale to better display microglia processes in white on black background. Scale bar in a-i is 25 µm. (**B**) Semi-quantitative analysis showed highest degree of OX-6 immunoexpression in ipsilateral striatum vs. control (baseline).

Analysis of activated microglia was also performed in the brains of tMCAO and control rats. A few OX-6 positive cells were identified in M1/M2 motor and secondary somatosensory cortices as well as striatum in controls (Figure 6A, a–c). However, a large number of activated microglial cells were determined in ipsilateral tMCAO hemisphere; in brain structures with EB extravasation (Figure 6A, d–f). The highest degree of OX-6 immunoexpression was determined in ipsilateral striatum (Figure 6B). Morphologically, these cells were characterized by large cell bodies and short processes. In the contralateral hemisphere, activated microglia were observed, mostly in the striatum and somatosensory cortex (Figure 6A, h, i), with a moderate level of OX-6 immunoreactivity (Figure 6B). Some OX-6 positive cells were also seen in the contralateral motor cortex (Figure 6A, g).

### Neuronal and Myelin Analyses

**Figure 7:**
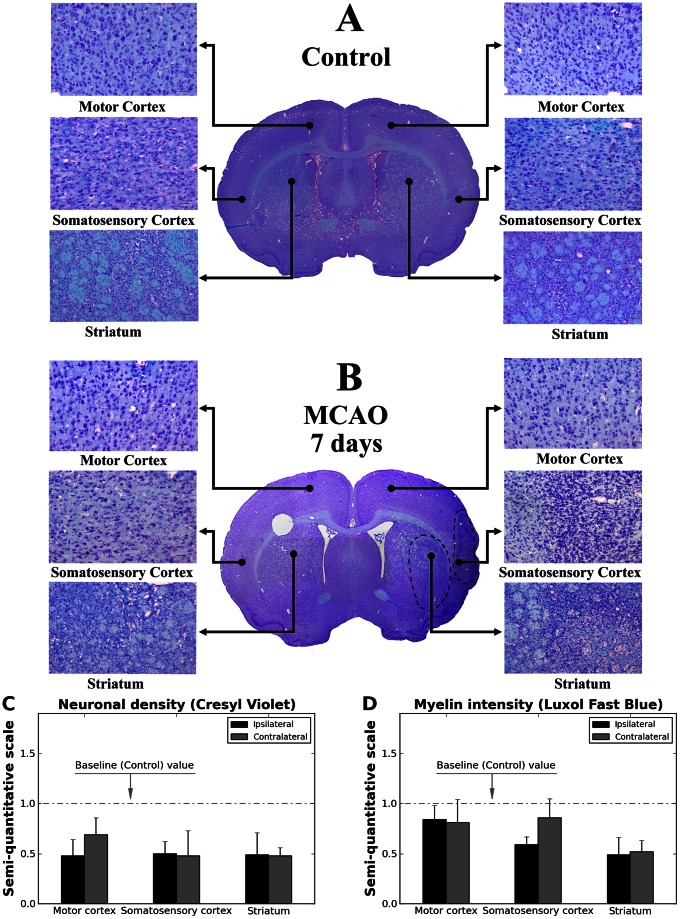
Histological analysis for neurons and myelin. Luxol Fast Blue-Cresyl Violet staining was performed in serial brain sections from control and MCAO rats for examination of myelin and neuron condition. (**A**) In controls, neurons were well established with distinct cell bodies and nuclei in examined cerebral structures from both hemispheres. Myelin also appeared normal. (**B**) In the brains of rats 7 days after MCAO, neuronal pyknosis and cellular debris were observed in the border zone of peri-infarct areas of striatum and somatosensory cortex in ipsilateral hemisphere. Fewer pyknotic neurons were seen in ipsilateral motor cortex, however, cell density was decreased compared to controls. Myelin thickness was also reduced. The lateral ventricle was expanded in the ipsilateral hemisphere. In the hemisphere contralateral to MCAO insult, fewer myelin sheaths were determined in somatosensory and motor cortices and striatum. Contralateral striatosomes also showed size decreases similar to striatosomes within infarct area. Neuronal density in motor and somatosensory cortices was diminished compared to controls. Some neurons appeared pyknotic. Scale bar in full brain images is 200 µm, in striatum is 100 µm, in motor and somatosensory cortices is 50 µm. (**C**) Semi-quantitative analysis of Cresyl Violet stained brain samples demonstrated reduction of neuronal densities in ipsilateral striatum, motor and somatosensory cortices. Neuronal densities were also diminished in analyzed cerebral cortices. Of note, neuronal cell densities in ipsilateral striatum and somatosensory cortex were analyzed outside of perilesional areas. (**D**) Semi-quantitative analysis of Luxol Fast Blue stained brain samples showed decrease of myelin intensity, mainly, in ipsilateral striatum and somatosensory cortex vs. control (baseline). Reduced myelin staining was also determined in contralateral striatum.

Luxol Fast Blue-Cresyl Violet staining was performed in serial brain sections from control and tMCAO rats for examination of myelin and neuron condition. In controls, neurons were easily determined, with distinct cell bodies and nuclei in examined cerebral structures of both hemispheres (Figure 7A). Myelin also appeared normal. Seven days after tMCAO, neuronal pyknosis and cellular debris were observed in the border zone of peri-infarct areas of striatum (CPu) and secondary somatosensory cortex in ipsilateral hemisphere (Figure 7B). Reductions in cell size and chromatin condensation were visible. Fewer pyknotic neurons were seen in ipsilateral motor cortex (M1/M2), however, cell density was decreased compared to controls (Figure 7C). Neuronal cell density was also reduced in ipsilateral striatum and somatosensory cortex outside of the perilesional areas (Figure 7C). Myelin thickness was abridged, typically, in these ipsilateral brain structures (Figure 7D). Additionally, the lateral ventricle was expanded in the ipsilateral hemisphere. In the hemisphere contralateral to tMCAO, fewer myelin sheaths were determined in somatosensory and motor cortices and the striatum (Figure 7B, D). Interestingly, contralateral striatosomes also showed size decreases similar to striatosomes within infarct area. Moreover, neuronal density in M1/M2 motor and secondary somatosensory cortices as well as in striatum (CPu) was diminished compared to controls (Figure 7C). Some neurons appeared pyknotic.

## Discussion

In the present study, we investigated subacute diaschisis in a focal ischemic stroke rat model. As expected, most damage was detected in the ipsilateral hemisphere, damage which included BBB alterations characterized by large EB parenchymal extravasation, autophagosome accumulation, increased reactive astrocytes and activated microglia, demyelinization, and neuronal damage in various brain structures 7 days after tMCAO. In parallel, BBB damage also occurred in the contralateral hemisphere. Major pathological changes in contralateral remote brain areas included (1) swollen and vacuolated endothelial cells containing numerous autophagosomes and enlarged mitochondria, (2) pericyte degeneration, (3) perivascular edema, (4) major EB extravasation, (5) increased autophagosome formation, (6) astrogliosis and appearance of activated microglia, (7) decreased myelin, (8) neuronal pyknosis. These pathological disturbances observed in contralateral remote brain areas suggest subacute diaschisis with a damaged BBB playing a major role.

The BBB impairment in ipsilateral and contralateral striatum and motor cortex determined at the ultrastructural level represents a main pathologic feature of subacute tMCAO. In the hemisphere ipsilateral to MCAO insult, microvascular injury was characterized by capillary EC damage and pericyte and astrocyte degeneration compromising BBB integrity. Edematous protein-filled perivascular areas populated by degenerating astrocyte cell processes were also observed via electron microscopy. Detection of a ruptured microaneurysm in the ipsilateral motor cortex confirmed vascular damage. This break in the microvascular wall might have been associated with the presence of thrombogenic cells, harbingers of blood clots and spontaneous hemorrhagic transformation. Indeed, hemorrhagic transformation has been implicated as a serious complication of ischemic stroke in that hemorrhagic transformation occurs in 12% of patients not receiving thrombolytic therapy and accompanies the progressive expansion of the ischemic area [50]. Mechanisms of hemorrhagic transformation in ischemic stroke are becoming recognized (reviewed in [51]), with a loss of basal lamina components shown to contribute to microvascular permeability and possibly leading to hemorrhagic consequences in ischemic stroke (reviewed in [24]). In our study, the appearance of a ruptured capillary 7 days after ischemic insult is likely due to prolonged EC pathology in addition to possible degradation of basement membrane components, possibilities currently under our investigation. Other important findings are the observed significant, although less severe, ultrastructural abnormalities in capillary EC in the hemisphere contralateral to tMCAO. Degenerated EC and EC with cytoplasmic vacuole formation and swollen mitochondria were seen. These vascular injuries in the hemisphere opposite to stroke insult may indicate pervasive BBB damage and ongoing pathological vascular changes in subacute phase of ischemia. Interestingly, Pillai et al. [52] demonstrated the relationship between BBB permeability and edema formation at different times after ischemia-reperfusion injury in rats using 3T MRI. Results showed that the first phase of the biphasic BBB opening lasted up to 24 hours after focal cerebral ischemia and was accompanied by progressive edema accumulation. In the second phase (48 hours post-reperfusion), BBB permeability did not lead to edema formation, suggesting vasogenic edema resorption. The authors also detected edema formation in the contralateral striatum at 4 hours post-reperfusion. Although these results showed the importance of BBB alterations in terms of local or remote edema formation, this study was limited to the acute ischemic phase. Further studies are needed to demonstrate BBB integrity in cerebral diaschisis at different ischemic stages. Currently, we are investigating vascular disturbances with a focus on diaschisis in chronic ischemic condition based on our findings in this study.

This widespread ultrastructural BBB alterations in subacute ischemia accompanies the vascular leakage, as confirmed by EB extravasation into the brain parenchyma. A significantly (5 times) higher level of extravasated EB was detected in the ipsilateral hemisphere compared to control. Although EB extravasation in the contralateral hemisphere was significantly less than in ipsilateral, it was still 3 times that of non-ischemic controls. In accordance with previous studies [9], [53]–[55], our results confirmed extensive vascular leakage at the ischemic injury site, but we noted an elevated level of EB extravasation in the contralateral hemisphere. Although mechanisms of BBB permeability at different phases of stroke are still poorly understood, this discrepancy might be due to pervasive BBB damage leading to vasogenic edema in the hemisphere opposite to stroke insult as demonstrated in our study. Neovascular permeability is another likely contributor to the increased level of EB extravasation. Moreover, in addition to increased paracellular permeability due to decreased tight junction integrity respective to the phases of ischemic stroke (reviewed in [56]), endothelial transcellular permeability should be considered since damaged capillary ECs were detected in our study of subacute ischemic insult in both ipsi- and contralateral hemispheres. Since our study is ongoing, the particular brain structures contributing to EB leakage in contralateral hemisphere will be investigated. However, it is clear that the BBB was still open 7 days after tMCAO and our data support previous studies showing BBB leakage for up to several weeks [20], [21]. This vascular leakage appeared limited to the brain, in that EB extravasation into parenchyma of the liver, an organ with highly fenestrated capillaries, revealed no significant differences in EB levels between control and MCAO rats.

Another interesting observation in the present study is the autophagosome accumulation within not only ipsilateral but also in contralateral capillary ECs. The essential role of autophagy is maintaining cell homeostasis by degradation of cytosolic components through an autophagosomal-lysosomal pathway [57]. The function of autophagy in stroke is complex. Removal of damaged cellular components and metabolic toxins promotes cell survival while excessive induced autophagy may degrade critical cell components and induce cell death [58]. Dramatic increases of autophagosomes in penumbra neurons have been shown beginning at 6 hours and lasting up to 48 hours post-ischemia in tMCAO rats through upregulation of Beclin-1 [59]. However, whether enhanced autophagy is part of cell survival or death in ischemia/reperfusion environment is still unclear. It has been suggested that activation of autophagy might partially protect neurons from ischemic insult and then autophagy might progress towards neuronal necrosis by building molecular blocks [60], [61]. Our study demonstrated, for the first time, excessive autophagosome accumulation within capillary ECs. Considering the dual role of autophagy in cell function maintenance, it is possible that autophagosomes play a protective role in EC survival in early stage acute post-ischemia. In subacute post-stroke condition, autophagy might assume a more deleterious role in ECs, even causing the cell necrosis as determined by EM analysis in capillaries of ipsilateral and, importantly, contralateral motor cortex and striatum. Although somatosensory cortex capillaries were not analyzed via EM, immunofluorescein Beclin-1 expression in this remote area confirmed significant autophagosome accumulation similar to that in striatum and motor cortex capillaries in ipsilateral hemisphere. These results, suggesting that EC autophagy might contribute to cell impairment and to BBB alteration and should be considered in subacute diaschisis, warrant further investigations. Of note, because metabolic disturbances were found in non-ischemic brain regions at 30 min post-MCAO in rats [62], it may be important to correlate metabolic levels in remote brain areas with EC autophagy.

Histological results showed rampant reactive astrocytes and activated microglia in remote striatum, motor and somatosensory cortices 7 days after tMCAO, likely indicating an inflammatory response. Inflammation is the main pathogenic event in ischemic stroke [63]–[65] and activation of these glial cells plays a major role in contributing and enhancing neuronal damage through secretion of various pro-inflammatory cytokines and chemokines. Moreover, inflammation closely interacts with other exacerbating factors, such as oxidative stress and the endothelial-matrix in ischemic vascular endothelial injury [66], [67], increasing BBB permeability [68]. The extent of the glial cell response: local, widespread, or systemic in subacute ischemic diaschisis needs to be clarified. However, a recent study [69] using a mouse tMCAO model demonstrated no obvious differences in astrocyte reactivity between lateral ventricular wall in whole mounts of MCAO contralateral hemispheres at 7 days post-stroke and sham-operated controls. Reactive astrogliosis in the subventrical zone (SVZ), disrupted neuroblast migratory scaffold, and ependymal cell alteration were detected in the ischemic lesion site. The authors noted that stroke induces multiple dramatic changes in the SVZ neurogenic compartment and that SVZ astrocytes might respond to the distant injury due to vascular-born or cerebral fluid (CSF)-born signals. Although there is a discrepancy between this study results and ours regarding astrogliosis in contralateral hemisphere at subacute tMCAO, the previous authors limited their investigation to brain areas adjacent to the lateral ventricular wall such as the overlying cerebral cortex and hippocampus. In addition to our observations of pathological BBB disturbances in contralateral striatum and cerebral cortex coupled with glial cell activation, it is possible that the blood-CSF barrier is also impaired and that this impairment may have contributed to the glial response in the contralateral hemisphere.

Additionally, in remote striatum, motor and somatosensory cortices, marked neuronal pyknosis was evident in subacute ischemia. This prolonged post-ischemia neuron damage may contribute to long-lasting stroke pathology and impede recovery processes. Interestingly, apoptotic cells were noted in hemisphere contralateral to initial insult of patients with acute stroke [70]. In a study by Dihné et al. [71] secondary neuronal damage and glial cell reactions were investigated in remote thalamic nuclei using two ischemic models in rats. The authors showed numerous damaged neurons within the ipsilateral ventroposterior and reticular thalamic nuclei compared to the contralateral side at 7 and 14 days after suture tMCAO. Astrocytic and microglial activation was also restricted to the ipsilateral thalamus and persisted up to 14 days after ischemia. Whereas in the photothrombotic ischemia, they detected delayed neuronal cell loss and glial activation only within the ipsilateral ventroposterior thalamic nucleus. The authors concluded that suture tMCAO, which resulted in widespread edema, might lead to secondary damage in examined thalamic nuclei, whereas photothrombotic ischemia characterized by pure cortical infarcts affected only the ventroposterior thalamic nucleus, likely due to retrograde degeneration of thalamocortical projections. Although these study results are important for understanding thalamic diaschisis, analysis of existing edema or retrograde transport was not performed in the thalamus. In our study, endothelial cell damage and perivascular edema were observed at ultrastructural level in both ipsilateral and contralateral hemispheres 7 days after tMCAO which might induce additional neuronal damage accompanied by glial cell activation.

Finally, diminished myelin was determined in brain structures with neuronal damage, primarily in the ipsilateral hemisphere. A partial reduction of myelin was noted in the contralateral hemisphere with pronounced decreases in striatosome size, similar to effects on striatosomes within the infarct area. Since communications between brain regions are essential to normal brain function, mechanisms contributing to white matter damage [8], [72]. Determining involvement of oligodendrocytes in myelin production at subacute ischemic phase might be essential for understanding possible damage to white matter tracts. Also, contralateral brain injury mediated via subacute diaschisis is likely induced from the initially acute ischemic brain areas through transneuronal pathways. Contralateral brain pathology might depend on the severity of acute ischemic damage. Furthermore, described pervasive BBB impairment in subacute phase of ischemia may indicate ongoing pathological vascular changes in association with neurodegenerative processes, possibly leading to development of cognitive decline and post-stroke dementia. More than 30% of patients who survived stroke attack developed dementia inside two years [73]. Although the pathogenesis of dementia is complex, vascular damage in post-stroke patients is one of the main risk factors, depending on the severity (volume and site) of cerebral insult and white matter injury [74]. However, how stroke might initiate neurodegenerative dementia, specifically in the aging population, is still under debate (reviewed in [75]–[77]).

In summary, subacute diaschisis was detected in a focal ischemic stroke rat model. We observed BBB breakdown and endothelial autophagosome accumulation in remote brain capillaries. This microvascular damage in subacute phase is closely associated with ischemic diaschisis, suggesting that subsequent repair of the injured vasculature proximal and distal to the primary ischemic brain site should be considered in the development of treatment strategies for stroke. One potential therapeutic approach is repair of the damaged BBB that could prevent further degeneration of surviving neurons. Recognizing that the BBB is a therapeutic “target” is important for developing neuroprotective strategies in stroke.
